# Supplementary material for: USA vs Russia in the scientific arena
Source: PLoS One. 2023 Jul 6;18(7):e0288152. doi: 10.1371/journal.pone.0288152 (PMC10325073; doi:10.1371/journal.pone.0288152)
Supplement: S2 Data — (PDF) [file pone.0288152.s002.pdf]

Data S1 (S1-Research_Staff_SC)
Research staff of the two countries in the 146 SC considered

Subject category	Area	Russia	United States
Physics, Applied	Physics	3440	5691
Biochemistry & Molecular Biology	Biology	2728	17382
Materials Science, Multidisciplinary	Engineering	2259	8423
Chemistry, Physical	Chemistry	2136	3970
Optics	Physics	2101	5056
Chemistry, Multidisciplinary	Chemistry	1902	12146
Physics, Condensed Matter	Physics	1859	929
Physics, Particles & Fields	Physics	1626	4379
Engineering, Electrical & Electronic	Engineering	1473	10452
Astronomy & Astrophysics	Physics	1103	7977
Chemistry, Organic	Chemistry	1021	1153
Geosciences, Multidisciplinary	Earth and Space Sciences	998	2887
Physics, Multidisciplinary	Physics	917	1006
Mathematics	Mathematics	889	3403
Chemistry, Inorganic & Nuclear	Chemistry	847	430
Metallurgy & Metallurgical Engineering	Engineering	787	235
Medicine, Research & Experimental	Biomedical Research	750	1790
Cardiac & Cardiovascular Systems	Clinical Medicine	749	12899
Geochemistry & Geophysics	Earth and Space Sciences	740	2812
Polymer Science	Chemistry	719	941
Mechanics	Physics	663	1152
Genetics & Heredity	Clinical Medicine	659	6108
Physics, Nuclear	Physics	548	1813
Physics, Fluids & Plasmas	Physics	544	1606
Neurosciences	Clinical Medicine	533	16456
Microbiology	Biology	528	5637
Plant Sciences	Biology	523	4938
Zoology	Biology	507	3228
Oncology	Biomedical Research	498	27160
Nuclear Science & Technology	Engineering	467	903
Meteorology & Atmospheric Sciences	Earth and Space Sciences	431	3776
Hematology	Biomedical Research	410	8359
Mathematics, Applied	Mathematics	405	1598
Chemistry, Analytical	Chemistry	399	1803
Engineering, Chemical	Engineering	386	1129
Computer Science, Theory & Methods	Engineering	344	3393
Instruments & Instrumentation	Engineering	328	507
Physics, Atomic, Molecular & Chemical	Physics	291	762
Materials Science, Ceramics	Engineering	287	167
Soil Science	Biology	276	410
Energy & Fuels	Physics	275	1679
Automation & Control Systems	Engineering	274	1124
Electrochemistry	Chemistry	273	485
Oceanography	Earth and Space Sciences	269	1540
Thermodynamics	Physics	265	324
Clinical Neurology	Clinical Medicine	264	12250
Chemistry, Applied	Chemistry	263	68
Ecology	Biology	262	6228
Environmental Sciences	Earth and Space Sciences	257	7061
Marine & Freshwater Biology	Biology	242	1865
Engineering, Mechanical	Engineering	236	1878
Crystallography	Physics	233	83
Computer Science, Artificial Intelligence	Engineering	229	2625
Cell Biology	Biology	224	9341

Pharmacology & Pharmacy	Biomedical Research	222	5238
Immunology	Biomedical Research	217	10099
Medicine, General & Internal	Clinical Medicine	206	3623
Respiratory System	Clinical Medicine	205	5252
Paleontology	Earth and Space Sciences	200	533
Rheumatology	Clinical Medicine	190	2506
Mineralogy	Earth and Space Sciences	188	96
Psychiatry	Clinical Medicine	178	6575
Endocrinology & Metabolism	Clinical Medicine	176	5726
Physics, Mathematical	Physics	174	533
Biotechnology & Applied Microbiology	Biology	157	1531
Chemistry, Medicinal	Biomedical Research	155	2395
Mining & Mineral Processing	Engineering	152	40
Peripheral Vascular Disease	Clinical Medicine	147	2220
Biophysics	Biology	143	2409
Biology	Biology	139	856
Acoustics	Physics	137	404
Spectroscopy	Physics	122	178
Telecommunications	Engineering	120	1226
Education & Educational Research	Law, political and social sciences	112	2071
Engineering, Aerospace	Engineering	111	966
Physiology	Clinical Medicine	109	662
Economics	Economics	103	4920
Virology	Biomedical Research	99	2879
Materials Science, Characterization & Testing	Engineering	93	40
Psychology, Multidisciplinary	Law, political and social sciences	93	1848
Radiology, Nuclear Medicine & Medical Imaging	Biomedical Research	91	8187
Water Resources	Earth and Space Sciences	89	1370
Infectious Diseases	Biomedical Research	89	4317
Gastroenterology & Hepatology	Clinical Medicine	85	7706
Urology & Nephrology	Clinical Medicine	82	4864
Sociology	Law, political and social sciences	81	1513
Nanoscience & Nanotechnology	Engineering	71	864
Statistics & Probability	Mathematics	69	1758
Computer Science, Information Systems	Engineering	64	1739
Engineering, Petroleum	Engineering	63	106
Management	Economics	60	1837
Engineering, Multidisciplinary	Engineering	53	132
Parasitology	Clinical Medicine	53	371
Remote Sensing	Engineering	53	997
Surgery	Clinical Medicine	53	10512
Engineering, Industrial	Engineering	52	184
Engineering, Civil	Engineering	51	1694
Entomology	Biology	49	1835
Computer Science, Software Engineering	Engineering	48	1249
Public, Environmental & Occupational Health	Clinical Medicine	46	11417
Mycology	Biology	45	154
Pathology	Biomedical Research	45	3376
Agriculture, Dairy & Animal Science	Biology	42	1909
Geology	Earth and Space Sciences	40	114
Transplantation	Clinical Medicine	35	1492
Business	Economics	35	1566
Obstetrics & Gynecology	Clinical Medicine	34	4655
Ophthalmology	Clinical Medicine	34	5670
Food Science & Technology	Biology	32	1400

Anthropology	Law, political and social sciences	31	1426
Social Sciences, Interdisciplinary	Law, political and social sciences	29	202
Biochemical Research Methods	Biology	29	934
Engineering, Biomedical	Engineering	29	2179
Computer Science, Interdisciplinary Applications	Engineering	28	413
Psychology, Educational	Psychology	27	556
Developmental Biology	Biology	27	1112
Engineering, Manufacturing	Engineering	26	292
Construction & Building Technology	Engineering	25	363
Engineering, Geological	Engineering	25	394
Pediatrics	Clinical Medicine	25	4421
Imaging Science & Photographic Technology	Physics	24	113
Forestry	Biology	24	896
Toxicology	Biomedical Research	24	2403
Dermatology	Clinical Medicine	24	3071
Fisheries	Biology	22	1252
Psychology, Experimental	Psychology	22	1638
Robotics	Engineering	18	805
Operations Research & Management Science	Mathematics	18	1043
Ornithology	Biology	17	367
Allergy	Biomedical Research	17	496
Transportation Science & Technology	Engineering	15	466
Materials Science, Textiles	Engineering	14	55
Geography, Physical	Earth and Space Sciences	14	74
Psychology	Psychology	14	483
Health Policy & Services	Law, political and social sciences	13	735
Materials Science, Biomaterials	Engineering	12	523
Materials Science, Coatings & Films	Engineering	11	60
Information Science & Library Science	Engineering	11	629
Health Care Sciences & Services	Clinical Medicine	11	3546
Veterinary Sciences	Biology	11	3566
Sport Sciences	Clinical Medicine	11	3723
Green & Sustainable Science & Technology	Earth and Space Sciences	10	28
Mathematics, Interdisciplinary Applications	Mathematics	10	156
Computer Science, Cybernetics	Engineering	10	220
Political Science	Law, political and social sciences	10	2000
Critical Care Medicine	Clinical Medicine	10	2502

Data S2 (S2-Research_Staff_Area)
Research staff of the two countries in the 11 area considered

Area	Russia	United States	World	Russia/United States	United States/World
Biology	6027	67250	317982	9.0%	21.1%
Biomedical Research	2617	76699	294088	3.4%	26.1%
Chemistry	7560	20996	144063	36.0%	14.6%
Clinical Medicine	3919	138227	520188	2.8%	26.6%
Earth and Space Sciences	3236	20291	120273	15.9%	16.9%
Economics	198	8323	30402	2.4%	27.4%
Engineering	8225	46472	367770	17.7%	12.6%
Law, political and social sciences	369	9795	28950	3.8%	33.8%
Mathematics	1391	7958	45815	17.5%	17.4%
Physics	14322	33685	203012	42.5%	16.6%
Psychology	63	2677	7396	2.4%	36.2%
Total	47927	432373	2079939	11.1%	20.8%

Data S3 (S3-TFI)
Total fractional impact of the two countries in the 146 SC considered

Subject category	Area	TFI Russia	TFI United States	TFI World total	World share Russia	World share United States	World rank Russia	World rank United States
Crystallography	Physics	243.244	233.777	3415.162	7.1%	6.8%	3	6
Chemistry, Inorganic & Nuclear	Chemistry	1463.965	992.885	24430.554	6.0%	4.1%	4	8
Engineering, Petroleum	Engineering	33.918	433.164	816.534	4.2%	53.0%	4	1
Materials Science, Characterization & Testing	Engineering	80.740	113.908	741.807	10.9%	15.4%	4	1
Metallurgy & Metallurgical Engineering	Engineering	581.527	663.905	9486.436	6.1%	7.0%	4	3
Mineralogy	Earth and Space Sciences	285.468	235.942	3036.963	9.4%	7.8%	4	6
Spectroscopy	Physics	223.610	389.109	3109.105	7.2%	12.5%	4	2
Engineering, Aerospace	Engineering	238.572	2024.428	7143.364	3.3%	28.3%	5	2
Mining & Mineral Processing	Engineering	111.353	104.576	1635.934	6.8%	6.4%	5	6
Optics	Physics	3303.323	16427.117	85991.736	3.8%	19.1%	5	2
Physics, Applied	Physics	4105.237	19088.109	85434.869	4.8%	22.3%	5	1
Physics, Condensed Matter	Physics	1819.975	4088.098	24922.051	7.3%	16.4%	5	1
Thermodynamics	Physics	650.474	1316.795	18611.090	3.5%	7.1%	5	3
Acoustics	Physics	157.039	955.180	4065.233	3.9%	23.5%	6	1
Chemistry, Applied	Chemistry	124.559	157.571	3431.567	3.6%	4.6%	6	4
Chemistry, Organic	Chemistry	1409.483	2208.757	33978.142	4.1%	6.5%	6	4
Mathematics	Mathematics	2300.934	10919.100	56450.002	4.1%	19.3%	6	1
Physics, Atomic, Molecular & Chemical	Physics	384.512	1879.566	9112.944	4.2%	20.6%	6	1
Physics, Fluids & Plasmas	Physics	804.264	4974.755	17020.873	4.7%	29.2%	6	1
Medicine, Research & Experimental	Biomedical Research	333.909	2739.507	10609.892	3.1%	25.8%	7	2
Physics, Multidisciplinary	Physics	1148.501	4487.037	33709.942	3.4%	13.3%	7	2
Physics, Nuclear	Physics	470.874	3179.613	12268.879	3.8%	25.9%	8	1
Materials Science, Ceramics	Engineering	214.457	513.383	6420.507	3.3%	8.0%	9	3
Paleontology	Earth and Space Sciences	355.653	1336.679	8134.705	4.4%	16.4%	9	1
Physics, Mathematical	Physics	505.271	2594.486	13630.313	3.7%	19.0%	9	1
Physics, Particles & Fields	Physics	1897.595	12206.707	54115.026	3.5%	22.6%	9	1
Zoology	Biology	819.526	6843.191	25562.310	3.2%	26.8%	9	1
Imaging Science & Photographic Technology	Physics	26.041	246.989	1560.985	1.7%	15.8%	10	2
Mechanics	Physics	972.655	5564.286	42103.499	2.3%	13.2%	10	2
Geochemistry & Geophysics	Earth and Space Sciences	614.902	9721.644	31948.484	1.9%	30.4%	11	1
Geology	Earth and Space Sciences	31.243	309.172	2465.907	1.3%	12.5%	11	2
Instruments & Instrumentation	Engineering	132.351	715.512	6596.639	2.0%	10.8%	11	3
Soil Science	Biology	256.964	1575.594	11262.112	2.3%	14.0%	11	2
Mathematics, Applied	Mathematics	1025.647	8820.397	62608.270	1.6%	14.1%	12	2
Nuclear Science & Technology	Engineering	268.038	1689.539	11437.023	2.3%	14.8%	12	1
Social Sciences, Interdisciplinary	Law, political and social sciences	30.430	514.880	1975.673	1.5%	26.1%	12	1
Astronomy & Astrophysics	Physics	1407.391	30028.597	86262.314	1.6%	34.8%	13	1
Chemistry, Physical	Chemistry	2623.241	16939.973	127279.612	2.1%	13.3%	13	2
Biochemistry & Molecular Biology	Biology	2494.955	41290.050	145727.626	1.7%	28.3%	14	1
Biophysics	Biology	105.390	4419.896	8676.425	1.2%	50.9%	14	1
Chemistry, Multidisciplinary	Chemistry	2251.941	43804.642	162616.637	1.4%	26.9%	14	1
Oceanography	Earth and Space Sciences	292.430	5327.425	14160.190	2.1%	37.6%	14	1
Geosciences, Multidisciplinary	Earth and Space Sciences	906.600	9426.511	55956.152	1.6%	16.8%	15	1
Electrochemistry	Chemistry	292.623	1801.737	15322.445	1.9%	11.8%	17	2
Materials Science, Multidisciplinary	Engineering	3247.616	49355.768	344576.622	0.9%	14.3%	18	2
Meteorology & Atmospheric Sciences	Earth and Space Sciences	359.639	14763.794	38803.442	0.9%	38.0%	18	1
Biology	Biology	48.875	1333.280	4434.462	1.1%	30.1%	19	1
Genetics & Heredity	Clinical Medicine	377.388	15560.894	44570.985	0.8%	34.9%	19	1
Automation & Control Systems	Engineering	465.867	7390.196	54043.442	0.9%	13.7%	20	2
Geography, Physical	Earth and Space Sciences	14.027	133.310	1181.671	1.2%	11.3%	20	4
Polymer Science	Chemistry	670.724	3643.337	48302.783	1.4%	7.5%	20	2
Sociology	Law, political and social sciences	48.654	5608.382	10769.733	0.5%	52.1%	20	1
Chemistry, Analytical	Chemistry	528.179	5036.481	50188.845	1.1%	10.0%	21	2
Mathematics, Interdisciplinary Applications	Mathematics	34.589	912.102	4486.484	0.8%	20.3%	21	2
Peripheral Vascular Disease	Clinical Medicine	121.674	5803.041	17773.551	0.7%	32.6%	21	1
Developmental Biology	Biology	17.807	1982.809	4395.185	0.4%	45.1%	22	1
Entomology	Biology	176.450	5549.428	17885.649	1.0%	31.0%	23	1
Marine & Freshwater Biology	Biology	233.164	4034.212	24594.858	0.9%	16.4%	23	1
Physiology	Clinical Medicine	61.357	1529.778	7224.002	0.8%	21.2%	23	1
Anthropology	Law, political and social sciences	38.491	2902.655	6349.259	0.6%	45.7%	24	1
Chemistry, Medicinal	Biomedical Research	162.066	3297.093	20481.499	0.8%	16.1%	24	2
Computer Science, Theory & Methods	Engineering	404.322	13523.729	49464.618	0.8%	27.3%	24	1
Microbiology	Biology	435.418	17660.013	62770.952	0.7%	28.1%	24	1
Mycology	Biology	40.098	393.833	4227.354	0.9%	9.3%	24	3
Ornithology	Biology	15.843	481.728	2024.781	0.8%	23.8%	24	1
Psychology	Psychology	2.769	1158.852	2009.761	0.1%	57.7%	24	1
Plant Sciences	Biology	571.289	12954.023	80105.941	0.7%	16.2%	26	1
Psychology, Educational	Psychology	9.811	1916.570	4393.648	0.2%	43.6%	26	1
Virology	Biomedical Research	56.601	6879.085	17143.378	0.3%	40.1%	26	1
Biochemical Research Methods	Biology	32.993	2611.004	8065.884	0.4%	32.4%	27	1
Engineering, Chemical	Engineering	346.408	3878.478	53695.644	0.6%	7.2%	27	2
Engineering, Mechanical	Engineering	163.689	5661.988	34272.323	0.5%	16.5%	27	2
Biotechnology & Applied Microbiology	Biology	185.123	5091.737	29816.264	0.6%	17.1%	28	1
Cell Biology	Biology	166.260	25403.454	62890.679	0.3%	40.4%	28	1
Business	Economics	66.469	4940.990	17381.835	0.4%	28.4%	29	1
Hematology	Biomedical Research	114.212	20834.985	50375.169	0.2%	41.4%	29	1
Materials Science, Coatings & Films	Engineering	7.581	80.090	1674.106	0.5%	4.8%	29	7
Nanoscience & Nanotechnology	Engineering	55.755	3270.083	13475.671	0.4%	24.3%	29	2
Neurosciences	Clinical Medicine	453.519	50794.585	135475.224	0.3%	37.5%	29	1
Political Science	Law, political and social sciences	28.297	7566.959	16321.685	0.2%	46.4%	29	1
Cardiac & Cardiovascular Systems	Clinical Medicine	285.157	35970.946	97886.875	0.3%	36.7%	30	1
Psychology, Experimental	Psychology	16.591	6322.241	17700.974	0.1%	35.7%	30	1
Robotics	Engineering	37.145	4526.345	16412.174	0.2%	27.6%	30	1
Telecommunications	Engineering	213.757	7867.463	54266.974	0.4%	14.5%	30	2

Computer Science, Interdisciplinary Applications	Engineering	23.355	1256.318	5693.038	0.4%	22.1%	31	1
Engineering, Electrical & Electronic	Engineering	1282.922	49218.846	296946.523	0.4%	16.6%	31	2
Forestry	Biology	44.915	2304.570	11321.956	0.4%	20.4%	31	1
Information Science & Library Science	Engineering	11.594	1000.567	4573.548	0.3%	21.9%	31	1
Management	Economics	101.187	6480.755	22875.232	0.4%	28.3%	31	1
Materials Science, Textiles	Engineering	5.901	116.075	2572.487	0.2%	4.5%	31	8
Parasitology	Clinical Medicine	63.749	812.053	9812.577	0.6%	8.3%	31	2
Operations Research & Management Science	Mathematics	57.678	3796.747	19336.799	0.3%	19.6%	32	1
Psychology, Multidisciplinary	Law, political and social sciences	62.145	5104.234	15643.499	0.4%	32.6%	32	1
Statistics & Probability	Mathematics	71.850	6326.471	18025.860	0.4%	35.1%	32	1
Computer Science, Cybernetics	Engineering	9.317	949.110	3726.300	0.3%	25.5%	33	1
Ecology	Biology	154.011	20701.813	65129.195	0.2%	31.8%	33	1
Education & Educational Research	Law, political and social sciences	67.190	6299.548	22657.452	0.3%	27.8%	34	1
Energy & Fuels	Physics	355.281	5511.478	67418.102	0.5%	8.2%	34	2
Engineering, Industrial	Engineering	23.509	542.573	5826.625	0.4%	9.3%	34	2
Oncology	Biomedical Research	283.549	76480.467	200134.003	0.1%	38.2%	34	1
Computer Science, Artificial Intelligence	Engineering	438.492	28556.759	133608.556	0.3%	21.4%	35	2
Transportation Science & Technology	Engineering	14.782	2368.599	7967.107	0.2%	29.7%	35	1
Fisheries	Biology	42.075	2566.225	13528.464	0.3%	19.0%	36	1
Remote Sensing	Engineering	40.762	3962.066	23891.083	0.2%	16.6%	36	2
Allergy	Biomedical Research	6.309	1112.507	3782.687	0.2%	29.4%	37	1
Engineering, Manufacturing	Engineering	23.099	1623.997	12615.592	0.2%	12.9%	37	2
Immunology	Biomedical Research	143.780	25871.844	68441.162	0.2%	37.8%	37	1
Radiology, Nuclear Medicine & Medical Imaging	Biomedical Research	38.079	19353.664	54168.523	0.1%	35.7%	37	1
Water Resources	Earth and Space Sciences	68.798	4813.886	23932.939	0.3%	20.1%	37	1
Economics	Economics	132.785	17350.284	47711.384	0.3%	36.4%	38	1
Engineering, Multidisciplinary	Engineering	21.576	317.527	4158.949	0.5%	7.6%	38	2
Clinical Neurology	Clinical Medicine	98.164	34125.791	95692.124	0.1%	35.7%	39	1
Materials Science, Biomaterials	Engineering	11.745	1883.357	10210.513	0.1%	18.4%	39	2
Respiratory System	Clinical Medicine	32.008	10788.255	33557.916	0.1%	32.1%	39	1
Computer Science, Software Engineering	Engineering	34.077	5594.134	21182.883	0.2%	26.4%	40	1
Engineering, Geological	Engineering	9.825	1510.074	11902.380	0.1%	12.7%	40	2
Ophthalmology	Clinical Medicine	26.842	12694.222	36667.165	0.1%	34.6%	40	1
Rheumatology	Clinical Medicine	37.057	6528.196	27222.860	0.1%	24.0%	40	1
Medicine, General & Internal	Clinical Medicine	68.238	9781.569	33518.325	0.2%	29.2%	41	1
Computer Science, Information Systems	Engineering	89.448	8081.380	40432.004	0.2%	20.0%	42	2
Urology & Nephrology	Clinical Medicine	23.611	13257.633	38408.072	0.1%	34.5%	42	1
Psychiatry	Clinical Medicine	60.624	22413.509	71502.074	0.1%	31.3%	43	1
Dermatology	Clinical Medicine	20.255	8058.292	27553.392	0.1%	29.2%	44	1
Endocrinology & Metabolism	Clinical Medicine	47.884	16418.886	58718.703	0.1%	28.0%	44	1
Obstetrics & Gynecology	Clinical Medicine	24.012	10725.378	31582.619	0.1%	34.0%	44	1
Pharmacology & Pharmacy	Biomedical Research	168.446	10226.691	71119.850	0.2%	14.4%	44	1
Transplantation	Clinical Medicine	3.789	2670.851	7075.479	0.1%	37.7%	44	1
Construction & Building Technology	Engineering	15.238	1618.541	11665.626	0.1%	13.9%	45	2
Engineering, Biomedical	Engineering	18.941	6287.892	20777.480	0.1%	30.3%	45	1
Sport Sciences	Clinical Medicine	11.439	7979.966	27823.187	0.0%	28.7%	45	1
Engineering, Civil	Engineering	58.150	6204.249	36853.892	0.2%	16.8%	46	2
Environmental Sciences	Earth and Space Sciences	310.532	24844.099	159694.889	0.2%	15.6%	47	2
Gastroenterology & Hepatology	Clinical Medicine	23.145	17217.708	58636.171	0.0%	29.4%	48	1
Toxicology	Biomedical Research	20.527	4089.558	14002.239	0.1%	29.2%	48	1
Pathology	Biomedical Research	8.270	7482.003	13576.196	0.1%	55.1%	50	1
Critical Care Medicine	Clinical Medicine	2.150	5127.467	13850.497	0.0%	37.0%	51	1
Surgery	Clinical Medicine	18.702	24307.281	59617.656	0.0%	40.8%	53	1
Agriculture, Dairy & Animal Science	Biology	17.871	4237.205	21227.529	0.1%	20.0%	54	1
Green & Sustainable Science & Technology	Earth and Space Sciences	3.927	67.372	2639.470	0.1%	2.6%	56	13
Health Policy & Services	Law, political and social sciences	1.213	1905.900	4247.410	0.0%	44.9%	57	1
Infectious Diseases	Biomedical Research	51.540	10912.561	37432.440	0.1%	29.2%	60	1
Pediatrics	Clinical Medicine	5.811	10011.362	24226.716	0.0%	41.3%	65	1
Veterinary Sciences	Biology	11.232	6363.171	28995.874	0.0%	21.9%	65	1
Food Science & Technology	Biology	31.989	4727.070	63912.171	0.1%	7.4%	66	3
Health Care Sciences & Services	Clinical Medicine	0.395	8000.562	18477.076	0.0%	43.3%	78	1
Public, Environmental & Occupational Health	Clinical Medicine	20.222	35820.527	87063.504	0.0%	41.1%	80	1

Data S4 (S4-FSS)
Fractional scientific strength of the two countries in the 146 SC considered

Subject category	Area	Russia		United States	
		Obs	FSS	Obs	FSS
Agriculture, Dairy & Animal Science	Biology	42	0.210	1909	1.094
Biochemical Research Methods	Biology	29	0.442	934	1.086
Biochemistry & Molecular Biology	Biology	2728	0.446	17382	1.157
Biology	Biology	139	0.227	856	1.008
Biophysics	Biology	143	0.423	2409	1.053
Biotechnology & Applied Microbiology	Biology	157	0.492	1531	1.387
Cell Biology	Biology	224	0.333	9341	1.218
Developmental Biology	Biology	27	0.439	1112	1.187
Ecology	Biology	262	0.178	6228	1.008
Entomology	Biology	49	1.589	1835	1.334
Fisheries	Biology	22	0.894	1252	0.958
Food Science & Technology	Biology	32	0.345	1400	1.164
Forestry	Biology	24	0.885	896	1.217
Marine & Freshwater Biology	Biology	242	0.466	1865	1.046
Microbiology	Biology	528	0.344	5637	1.308
Mycology	Biology	45	0.375	154	1.075
Ornithology	Biology	17	0.583	367	0.821
Plant Sciences	Biology	523	0.505	4938	1.213
Soil Science	Biology	276	0.336	410	1.386
Veterinary Sciences	Biology	11	0.685	3566	1.198
Zoology	Biology	507	0.775	3228	1.016
Allergy	Biomedical Research	17	0.216	496	1.307
Chemistry, Medicinal	Biomedical Research	155	0.576	2395	0.759
Hematology	Biomedical Research	410	0.167	8359	1.491
Immunology	Biomedical Research	217	0.318	10099	1.231
Infectious Diseases	Biomedical Research	89	0.321	4317	1.402
Medicine, Research & Experimental	Biomedical Research	750	0.361	1790	1.243
Oncology	Biomedical Research	498	0.259	27160	1.280
Pathology	Biomedical Research	45	0.105	3376	1.262
Pharmacology & Pharmacy	Biomedical Research	222	0.369	5238	0.950
Radiology, Nuclear Medicine & Medical Imaging	Biomedical Research	91	0.204	8187	1.152
Toxicology	Biomedical Research	24	0.507	2403	1.009
Virology	Biomedical Research	99	0.301	2879	1.256
Chemistry, Analytical	Chemistry	399	0.419	1803	0.884
Chemistry, Applied	Chemistry	263	0.223	68	1.092
Chemistry, Inorganic & Nuclear	Chemistry	847	0.588	430	0.786
Chemistry, Multidisciplinary	Chemistry	1902	0.331	12146	1.009
Chemistry, Organic	Chemistry	1021	0.548	1153	0.761
Chemistry, Physical	Chemistry	2136	0.351	3970	1.219
Electrochemistry	Chemistry	273	0.365	485	1.266
Polymer Science	Chemistry	719	0.286	941	1.189
Cardiac & Cardiovascular Systems	Clinical Medicine	749	0.190	12899	1.389
Clinical Neurology	Clinical Medicine	264	0.168	12250	1.259
Critical Care Medicine	Clinical Medicine	10	0.095	2502	0.906
Dermatology	Clinical Medicine	24	0.391	3071	1.217
Endocrinology & Metabolism	Clinical Medicine	176	0.123	5726	1.295
Gastroenterology & Hepatology	Clinical Medicine	85	0.136	7706	1.113
Genetics & Heredity	Clinical Medicine	659	0.306	6108	1.362
Health Care Sciences & Services	Clinical Medicine	11	0.015	3546	0.953
Medicine, General & Internal	Clinical Medicine	206	0.176	3623	1.434
Neurosciences	Clinical Medicine	533	0.341	16456	1.239
Obstetrics & Gynecology	Clinical Medicine	34	0.363	4655	1.186
Ophthalmology	Clinical Medicine	34	0.384	5670	1.088
Parasitology	Clinical Medicine	53	0.682	371	1.241
Pediatrics	Clinical Medicine	25	0.141	4421	1.373
Peripheral Vascular Disease	Clinical Medicine	147	0.408	2220	1.289
Physiology	Clinical Medicine	109	0.332	662	1.361

Psychiatry	Clinical Medicine	178	0.118	6575	1.184
Public, Environmental & Occupational Health	Clinical Medicine	46	0.168	11417	1.197
Respiratory System	Clinical Medicine	205	0.089	5252	1.175
Rheumatology	Clinical Medicine	190	0.100	2506	1.336
Sport Sciences	Clinical Medicine	11	0.401	3723	0.826
Surgery	Clinical Medicine	53	0.196	10512	1.285
Transplantation	Clinical Medicine	35	0.080	1492	1.319
Urology & Nephrology	Clinical Medicine	82	0.150	4864	1.418
Environmental Sciences	Earth and Space Sciences	257	0.387	7061	1.127
Geochemistry & Geophysics	Earth and Space Sciences	740	0.308	2812	1.280
Geography, Physical	Earth and Space Sciences	14	0.526	74	0.945
Geology	Earth and Space Sciences	40	0.341	114	1.184
Geosciences, Multidisciplinary	Earth and Space Sciences	998	0.360	2887	1.294
Green & Sustainable Science & Technology	Earth and Space Sciences	10	0.146	28	0.892
Meteorology & Atmospheric Sciences	Earth and Space Sciences	431	0.271	3776	1.269
Mineralogy	Earth and Space Sciences	188	0.761	96	1.231
Oceanography	Earth and Space Sciences	269	0.420	1540	1.337
Paleontology	Earth and Space Sciences	200	0.664	533	0.937
Water Resources	Earth and Space Sciences	89	0.257	1370	1.169
Business	Economics	35	0.587	1566	0.976
Economics	Economics	103	0.455	4920	1.244
Management	Economics	60	0.532	1837	1.114
Automation & Control Systems	Engineering	274	0.298	1124	1.151
Computer Science, Artificial Intelligence	Engineering	229	0.316	2625	1.796
Computer Science, Cybernetics	Engineering	10	0.280	220	1.298
Computer Science, Information Systems	Engineering	64	0.411	1739	1.367
Computer Science, Interdisciplinary Applications	Engineering	28	0.355	413	1.296
Computer Science, Software Engineering	Engineering	48	0.191	1249	1.204
Computer Science, Theory & Methods	Engineering	344	0.426	3393	1.445
Construction & Building Technology	Engineering	25	0.166	363	1.212
Engineering, Aerospace	Engineering	111	0.806	966	0.786
Engineering, Biomedical	Engineering	29	0.273	2179	1.206
Engineering, Chemical	Engineering	386	0.277	1129	1.060
Engineering, Civil	Engineering	51	0.328	1694	1.054
Engineering, Electrical & Electronic	Engineering	1473	0.249	10452	1.347
Engineering, Geological	Engineering	25	0.105	394	1.024
Engineering, Industrial	Engineering	52	0.138	184	0.903
Engineering, Manufacturing	Engineering	26	0.231	292	1.448
Engineering, Mechanical	Engineering	236	0.257	1878	1.119
Engineering, Multidisciplinary	Engineering	53	0.192	132	1.136
Engineering, Petroleum	Engineering	63	0.218	106	1.652
Information Science & Library Science	Engineering	11	0.343	629	0.518
Instruments & Instrumentation	Engineering	328	0.270	507	0.945
Materials Science, Biomaterials	Engineering	12	0.332	523	1.222
Materials Science, Ceramics	Engineering	287	0.323	167	1.330
Materials Science, Characterization & Testing	Engineering	93	0.518	40	1.701
Materials Science, Coatings & Films	Engineering	11	0.293	60	0.567
Materials Science, Multidisciplinary	Engineering	2259	0.353	8423	1.437
Materials Science, Textiles	Engineering	14	0.220	55	1.101
Metallurgy & Metallurgical Engineering	Engineering	787	0.419	235	1.604
Mining & Mineral Processing	Engineering	152	0.402	40	1.435
Nanoscience & Nanotechnology	Engineering	71	0.264	864	1.271
Nuclear Science & Technology	Engineering	467	0.355	903	1.157
Remote Sensing	Engineering	53	0.209	997	1.078
Robotics	Engineering	18	0.483	805	1.315
Telecommunications	Engineering	120	0.329	1226	1.185
Transportation Science & Technology	Engineering	15	0.267	466	1.379
Anthropology	Law, political and social sciences	31	0.591	1426	0.968
Education & Educational Research	Law, political and social sciences	112	0.220	2071	1.117

Health Policy & Services	Law, political and social sciences	13	0.041	735	1.129
Political Science	Law, political and social sciences	10	0.712	2000	0.952
Psychology, Multidisciplinary	Law, political and social sciences	93	0.259	1848	1.069
Social Sciences, Interdisciplinary	Law, political and social sciences	29	0.482	202	1.171
Sociology	Law, political and social sciences	81	0.168	1513	1.039
Mathematics	Mathematics	889	0.843	3403	1.045
Mathematics, Applied	Mathematics	405	0.532	1598	1.160
Mathematics, Interdisciplinary Applications	Mathematics	10	0.842	156	1.423
Operations Research & Management Science	Mathematics	18	0.835	1043	0.948
Statistics & Probability	Mathematics	69	0.386	1758	1.334
Acoustics	Physics	137	0.522	404	1.078
Astronomy & Astrophysics	Physics	1103	0.415	7977	1.223
Crystallography	Physics	233	0.582	83	1.569
Energy & Fuels	Physics	275	0.360	1679	0.915
Imaging Science & Photographic Technology	Physics	24	0.525	113	1.057
Mechanics	Physics	663	0.336	1152	1.105
Optics	Physics	2101	0.548	5056	1.132
Physics, Applied	Physics	3440	0.503	5691	1.414
Physics, Atomic, Molecular & Chemical	Physics	291	0.619	762	1.156
Physics, Condensed Matter	Physics	1859	0.449	929	2.020
Physics, Fluids & Plasmas	Physics	544	0.600	1606	1.257
Physics, Mathematical	Physics	174	0.797	533	1.336
Physics, Multidisciplinary	Physics	917	0.362	1006	1.289
Physics, Nuclear	Physics	548	0.552	1813	1.126
Physics, Particles & Fields	Physics	1626	0.487	4379	1.164
Spectroscopy	Physics	122	1.039	178	1.239
Thermodynamics	Physics	265	0.524	324	0.868
Psychology	Psychology	14	0.089	483	1.082
Psychology, Educational	Psychology	27	0.091	556	0.862
Psychology, Experimental	Psychology	22	0.222	1638	1.137

Data S5 (S5-SI)
Specialization indexes of the two countries in the 146 SC considered

Subject category	Area	Research staff			Specialization index	
		Russia	United States	World	Russia	United States
Agriculture, Dairy & Animal Science	Biology	42	1909	10854	0.168	0.846
Biochemical Research Methods	Biology	29	934	3182	0.396	1.412
Biochemistry & Molecular Biology	Biology	2728	17382	72657	1.629	1.151
Biology	Biology	139	856	2999	2.011	1.373
Biophysics	Biology	143	2409	5129	1.210	2.259
Biotechnology & Applied Microbiology	Biology	157	1531	12716	0.536	0.579
Cell Biology	Biology	224	9341	28681	0.339	1.567
Developmental Biology	Biology	27	1112	3019	0.388	1.772
Ecology	Biology	262	6228	20024	0.568	1.496
Entomology	Biology	49	1835	8075	0.263	1.093
Fisheries	Biology	22	1252	6443	0.148	0.935
Food Science & Technology	Biology	32	1400	22310	0.062	0.302
Forestry	Biology	24	896	5456	0.191	0.790
Marine & Freshwater Biology	Biology	242	1865	12108	0.867	0.741
Microbiology	Biology	528	5637	26588	0.862	1.020
Mycology	Biology	45	154	1826	1.070	0.406
Ornithology	Biology	17	367	1308	0.564	1.350
Plant Sciences	Biology	523	4938	37784	0.601	0.629
Soil Science	Biology	276	410	4147	2.888	0.476
Veterinary Sciences	Biology	11	3566	20134	0.024	0.852
Zoology	Biology	507	3228	12542	1.754	1.238
Allergy	Biomedical Research	17	496	2420	0.305	0.986
Chemistry, Medicinal	Biomedical Research	155	2395	11525	0.584	1.000
Hematology	Biomedical Research	410	8359	32239	0.552	1.247
Immunology	Biomedical Research	217	10099	33900	0.278	1.433
Infectious Diseases	Biomedical Research	89	4317	21114	0.183	0.984
Medicine, Research & Experimental	Biomedical Research	750	1790	8897	3.658	0.968
Oncology	Biomedical Research	498	27160	94564	0.229	1.382
Pathology	Biomedical Research	45	3376	8298	0.235	1.957
Pharmacology & Pharmacy	Biomedical Research	222	5238	35605	0.271	0.708
Radiology, Nuclear Medicine & Medical Imaging	Biomedical Research	91	8187	27703	0.143	1.422
Toxicology	Biomedical Research	24	2403	8628	0.121	1.340
Virology	Biomedical Research	99	2879	9195	0.467	1.506
Chemistry, Analytical	Chemistry	399	1803	16184	1.070	0.536
Chemistry, Applied	Chemistry	263	68	1675	6.814	0.195
Chemistry, Inorganic & Nuclear	Chemistry	847	430	8487	4.331	0.244
Chemistry, Multidisciplinary	Chemistry	1902	12146	46541	1.774	1.255
Chemistry, Organic	Chemistry	1021	1153	13859	3.197	0.400
Chemistry, Physical	Chemistry	2136	3970	36880	2.514	0.518
Electrochemistry	Chemistry	273	485	5316	2.229	0.439
Polymer Science	Chemistry	719	941	15121	2.064	0.299
Cardiac & Cardiovascular Systems	Clinical Medicine	749	12899	51503	0.631	1.205
Clinical Neurology	Clinical Medicine	264	12250	45075	0.254	1.307
Critical Care Medicine	Clinical Medicine	10	2502	6526	0.067	1.844
Dermatology	Clinical Medicine	24	3071	13403	0.078	1.102
Endocrinology & Metabolism	Clinical Medicine	176	5726	27511	0.278	1.001
Gastroenterology & Hepatology	Clinical Medicine	85	7706	31175	0.118	1.189
Genetics & Heredity	Clinical Medicine	659	6108	24289	1.177	1.210
Health Care Sciences & Services	Clinical Medicine	11	3546	8147	0.059	2.094
Medicine, General & Internal	Clinical Medicine	206	3623	18990	0.471	0.918
Neurosciences	Clinical Medicine	533	16456	55456	0.417	1.427
Obstetrics & Gynecology	Clinical Medicine	34	4655	17132	0.086	1.307
Ophthalmology	Clinical Medicine	34	5670	18544	0.080	1.471
Parasitology	Clinical Medicine	53	371	5649	0.407	0.316
Pediatrics	Clinical Medicine	25	4421	15402	0.070	1.381
Peripheral Vascular Disease	Clinical Medicine	147	2220	9290	0.687	1.150
Physiology	Clinical Medicine	109	662	4401	1.075	0.724
Psychiatry	Clinical Medicine	178	6575	25744	0.300	1.229
Public, Environmental & Occupational Health	Clinical Medicine	46	11417	33882	0.059	1.621
Respiratory System	Clinical Medicine	205	5252	20537	0.433	1.230
Rheumatology	Clinical Medicine	190	2506	14982	0.550	0.805
Sport Sciences	Clinical Medicine	11	3723	11139	0.043	1.608
Surgery	Clinical Medicine	53	10512	34732	0.066	1.456
Transplantation	Clinical Medicine	35	1492	5635	0.270	1.274
Urology & Nephrology	Clinical Medicine	82	4864	21044	0.169	1.112
Environmental Sciences	Earth and Space Sciences	257	7061	51790	0.215	0.656
Geochemistry & Geophysics	Earth and Space Sciences	740	2812	12062	2.662	1.121
Geography, Physical	Earth and Space Sciences	14	74	633	0.960	0.562
Geology	Earth and Space Sciences	40	114	1114	1.558	0.492

Geosciences, Multidisciplinary	Earth and Space Sciences	998	2887	22546	1.921	0.616
Green & Sustainable Science & Technology	Earth and Space Sciences	10	28	984	0.441	0.137
Meteorology & Atmospheric Sciences	Earth and Space Sciences	431	3776	12790	1.462	1.420
Mineralogy	Earth and Space Sciences	188	96	1562	5.223	0.296
Oceanography	Earth and Space Sciences	269	1540	5565	2.098	1.331
Paleontology	Earth and Space Sciences	200	533	3092	2.807	0.829
Water Resources	Earth and Space Sciences	89	1370	8135	0.475	0.810
Business	Economics	35	1566	5528	0.275	1.363
Economics	Economics	103	4920	17449	0.256	1.356
Management	Economics	60	1837	7425	0.351	1.190
Automation & Control Systems	Engineering	274	1124	9642	1.233	0.561
Computer Science, Artificial Intelligence	Engineering	229	2625	22450	0.443	0.562
Computer Science, Cybernetics	Engineering	10	220	1133	0.383	0.934
Computer Science, Information Systems	Engineering	64	1739	12160	0.228	0.688
Computer Science, Interdisciplinary Applications	Engineering	28	413	2504	0.485	0.793
Computer Science, Software Engineering	Engineering	48	1249	5788	0.360	1.038
Computer Science, Theory & Methods	Engineering	344	3393	18281	0.817	0.893
Construction & Building Technology	Engineering	25	363	3231	0.336	0.540
Engineering, Aerospace	Engineering	111	966	2808	1.716	1.655
Engineering, Biomedical	Engineering	29	2179	8868	0.142	1.182
Engineering, Chemical	Engineering	386	1129	16852	0.994	0.322
Engineering, Civil	Engineering	51	1694	10856	0.204	0.751
Engineering, Electrical & Electronic	Engineering	1473	10452	86680	0.737	0.580
Engineering, Geological	Engineering	25	394	3237	0.335	0.586
Engineering, Industrial	Engineering	52	184	1827	1.235	0.484
Engineering, Manufacturing	Engineering	26	292	3351	0.337	0.419
Engineering, Mechanical	Engineering	236	1878	13050	0.785	0.692
Engineering, Multidisciplinary	Engineering	53	132	2033	1.131	0.312
Engineering, Petroleum	Engineering	63	106	344	7.948	1.482
Information Science & Library Science	Engineering	11	629	1768	0.270	1.711
Instruments & Instrumentation	Engineering	328	507	4568	3.116	0.534
Materials Science, Biomaterials	Engineering	12	523	3489	0.149	0.721
Materials Science, Ceramics	Engineering	287	167	2870	4.340	0.280
Materials Science, Characterization & Testing	Engineering	93	40	481	8.391	0.400
Materials Science, Coatings & Films	Engineering	11	60	731	0.653	0.395
Materials Science, Multidisciplinary	Engineering	2259	8423	85883	1.142	0.472
Materials Science, Textiles	Engineering	14	55	1375	0.442	0.192
Metallurgy & Metallurgical Engineering	Engineering	787	235	5816	5.872	0.194
Mining & Mineral Processing	Engineering	152	40	922	7.155	0.209
Nanoscience & Nanotechnology	Engineering	71	864	4575	0.674	0.908
Nuclear Science & Technology	Engineering	467	903	7345	2.759	0.591
Remote Sensing	Engineering	53	997	6568	0.350	0.730
Robotics	Engineering	18	805	3884	0.201	0.997
Telecommunications	Engineering	120	1226	10203	0.510	0.578
Transportation Science & Technology	Engineering	15	466	2197	0.296	1.020
Anthropology	Law, political and social sciences	31	1426	3296	0.408	2.081
Education & Educational Research	Law, political and social sciences	112	2071	8721	0.557	1.142
Health Policy & Services	Law, political and social sciences	13	735	1992	0.283	1.775
Political Science	Law, political and social sciences	10	2000	4391	0.099	2.191
Psychology, Multidisciplinary	Law, political and social sciences	93	1848	6284	0.642	1.415
Social Sciences, Interdisciplinary	Law, political and social sciences	29	202	987	1.275	0.985
Sociology	Law, political and social sciences	81	1513	3279	1.072	2.220
Mathematics	Mathematics	889	3403	19207	2.009	0.852
Mathematics, Applied	Mathematics	405	1598	13499	1.302	0.569
Mathematics, Interdisciplinary Applications	Mathematics	10	156	1115	0.389	0.673
Operations Research & Management Science	Mathematics	18	1043	5133	0.152	0.977
Statistics & Probability	Mathematics	69	1758	6861	0.436	1.233
Acoustics	Physics	137	404	1904	3.123	1.021
Astronomy & Astrophysics	Physics	1103	7977	28425	1.684	1.350
Crystallography	Physics	233	83	1994	5.071	0.200
Energy & Fuels	Physics	275	1679	18973	0.629	0.426
Imaging Science & Photographic Technology	Physics	24	113	788	1.322	0.690
Mechanics	Physics	663	1152	9819	2.930	0.564
Optics	Physics	2101	5056	30612	2.979	0.795
Physics, Applied	Physics	3440	5691	36727	4.065	0.745
Physics, Atomic, Molecular & Chemical	Physics	291	762	4365	2.893	0.840
Physics, Condensed Matter	Physics	1859	929	11771	6.854	0.380
Physics, Fluids & Plasmas	Physics	544	1606	6984	3.380	1.106
Physics, Mathematical	Physics	174	533	3841	1.966	0.668
Physics, Multidisciplinary	Physics	917	1006	10013	3.974	0.483
Physics, Nuclear	Physics	548	1813	8100	2.936	1.077

Physics, Particles & Fields	Physics	1626	4379	22817	3.093	0.923
Spectroscopy	Physics	122	178	1820	2.909	0.470
Thermodynamics	Physics	265	324	4059	2.833	0.384
Psychology	Psychology	14	483	957	0.635	2.428
Psychology, Educational	Psychology	27	556	1136	1.031	2.354
Psychology, Experimental	Psychology	22	1638	5303	0.180	1.486
	Overall	47927	432373	2079939		
