# Supplementary material for: USA vs Russia in the scientific arena
Source: PLoS One. 2023 Jul 6;18(7):e0288152. doi: 10.1371/journal.pone.0288152 (PMC10325073; doi:10.1371/journal.pone.0288152)
Supplement: Attachment — Submitted filename: Reply by the authors.docx [file pone.0288152.s003.docx]

**REPLY BY THE AUTHORS**

**Reviewer #1**

This paper is well done, it is brave in its object of analysis, and it achieves its goals with a conscientious methodology. I think the paper should not be published before a number of (minor) issues be attended to.

Reply by the authors

We thank the reviewer for his appreciation. We tried to follow up on all his comments and revise the manuscript accordingly.

Some notes:

p. 3 – basic details about sources/metrics/indicators could perhaps be nailed in a minimalist but effective section that can help the reader in moving forward without having to jump to the “Materials” section below …

Reply by the authors

We have anticipated the Materials and Methods section.

p. 3 – in the “Results” section you refer to science staff USA vs RU is 9:1. All right, however:

- you should report the source (but note these data is not coming from WoS, so where does it come from?) and put out a word regarding comparability (the science systems are quite different; plus, there devilish details, e.g., how is university staff counted in these statistics? how are corporate researchers counted? etc.)

Reply by the authors

By research staff we mean the authors of publications indexed in WoS. The methodology to identify those authors is presented at the very beginning of the Materials and Methods section. We added a footnote to make it more clear: “The adopted methodology excludes from the counting of research staff researchers who did not publish in journals indexed in WoS.” To conduct our analyses we need names and field of research of each scientist. No databases containing that information are available. Consequently, we had to devise the above said methodology to extract the data.

- could you situate the reader regarding the relative sizes of the country populations, GDPs, absolute R&D figures? … note that the US is about 14x the size of the ruskie economy … this book is old, but gives some comparative perspective <https://books.google.pt/books?hl=pt-PT&lr=&id=T4doAgAAQBAJ&oi=fnd&pg=PP1&dq=size+of+US+and+russian+economies&ots=7SHXOBUnMt&sig=GSKBDHKhZcP-QT7scNTdjCZNiuo&redir_esc=y#v=onepage&q=size%20of%20US%20and%20russian%20economies&f=false>

Reply by the authors

We reported a few statistics about the two countries in the Conclusions section.

p. 4 – Regarding research productivity: we perhaps should not forget that US researchers publish directly in the global language (today’s latin) and that they control the outlets (see the recently growing literature on “editormetrics”, e.g. https://link.springer.com/article/10.1007/s11192-022-04279-9 and <https://link.springer.com/article/10.1007/s11192-017-2434-7>)

Reply by the authors

We agree. In fact, in the Conclusions section we state that “the 2021 Journal Citation Index covers 5930 journals published in the USA compared to 390 in Russia. Since roughly 2010, Russian researchers have had strong and increasing incentives to publish in indexed journals. However, there could be many who still publish in non-indexed, solely Russian-language journals.”

p. 5 – for sure in terms of efficiency something of the kind seems to be going on in terms of military spending … the US military-industrial complex is too expensive (costs in excess to the original procurement contracts, one thinks of the F35) for the results it delivers on the battle ground while russian gear appears to be quite sturdy (lighter in logistics, less needed of repairs) … see <https://link.springer.com/chapter/10.1057/9781403983428_8>

Reply by the authors

We trust the reviewer but, frankly speaking, we are not competent in military issues. The suggested publication dates 2006 and we do not know whether any changes have occurred ever since. We prefer to avoid entering a field we are not competent in.

p. 6 – I feel it would be useful to have a table summarising the key findings and highlighting the key knowledge fields that were reported. For instance, this papefr provides an important and timely antidote to the superficial talk on Russia as a country dependent on natural resources (science indicators show that this country does a lot to develop frontier knowledge about minerals, etc.). Any changes that the authors could do to favour the papers usability would be welcome and would leverage the impact of this work, which I would wish should be big.

Note: I wonder if this paper would not be useful in any way

https://direct.mit.edu/qss/article/3/1/37/109076/Scopus-1900-2020-Growth-in-articles-abstracts

Reply by the authors

We have integrated the paper with a concluding section where we wrap up the main findings and possible interpretations. We are somewhat hesitant to extend further their implications, as we are essentially bibliometricians, with no competencies in international policies and the like.

**Reviewer #2**

This work compares the scientific output between USA and Russia in 146 scientific disciplines. The results show that USA outperforms Russia in world impact. However, USA is less efficient in allocating resources to the disciplines where it performs better.

Some general comments

Reply by the authors

We wish to thank the reviewer for his valuable comments and suggestions. Our detailed feedback to each individual comment follows.

The "Materials and Methods" section should be before the "Winning the competition/Results" after the "Introduction" section. You are mentioning and analyzing relevant measures in your methodology, such as FSS, and only explain what they are at the end of the article. Also, there should be a section with Conclusions/Discussion regarding the work developed.

Reply by the authors

We have anticipated the Materials and methods section and integrated the paper with a concluding section.

The "Materials and Methods" should have a diagram workflow for the data selection and refinement. Also, specify the period considered (2015-2019) in this diagram/section.

Reply by the authors

Done

In Figure 1, the x-axis should have a label. From the text, it is easy to understand that each point represents one of the 146 of the SC, but by analyzing the plot, it is not straightforward. Also, in that case, you aim to plot a categorical variable against a continuous variable. The most appropriate graph for that is not a scatter plot, but a side-by-side boxplot. Since you have a lot of SC categories and areas, you could show the side-by-side for the overall measures and comment on the differences between areas in the text.

Reply by the authors

Done

Additionally, the information in Table 2 can be represented through a barplot (x- area and y-FSS) per country. The same thing applies to Table 1.

Reply by the authors

We agree with the reviewer’s suggestion concerning Table 2 (turned into Table 3 after the revision), but not for Table 1 since it shows different descriptive statistics that would be difficult to condense into a single figure.

**Reviewer #3**

The topic is actually interesting and developed at a time when everyone is looking for topics about Russia. The research is well-described, and the findings are extremely useful. For these reasons, I believe the manuscript can be published, but it requires major revisions and reorganization.

Reply by the authors

We thank the reviewer for his appreciation. We tried to follow up on all his comments and revise the manuscript accordingly.

It surprised me to see the results section before the materials and methods section. That should be changed so that readers can better understand the results. For example, the results discuss the "distribution of share of scholarly impact" and refer to TFI in brackets. However, it has not yet been defined, making it difficult to comprehend.

All previously used and defined short expression forms are defined again in the material and methods section. SC, for example, is defined twice in this section. Furthermore, "The field of observation then includes 146 SCs grouped in 11 areas" appears to be a new sentence that should follow a full stop.

Reply by the authors

We have solved this, anticipating the Materials and methods section.

Although the Gini coefficient is well known, it would benefit from a definition or reference.

Reply by the authors

We have defined the Gini coefficient.

Finally, I haven't come across any discussion or conclusion sections, and the paper would benefit from them.

Reply by the authors

We have integrated the paper with a concluding section.
